# EHMTI-0364. Non-invasive vagus nerve stimulation using gammacore® for prevention and acute treatment of chronic cluster headache: report from the randomized phase of the preva study

**DOI:** 10.1186/1129-2377-15-S1-I7

**Published:** 2014-09-18

**Authors:** C Gaul, H Diener, K Solbach, N Silver, A Straube, D Magis, U Reuter, A Andersson, EJ Liebler

**Affiliations:** 1 Migraine and Headache Clinic, University of Duisburg-Essen, Königstein, Germany; 2 Department of Neurology, University Hospital Essen, Essen, Germany; 3 Department of Neurology, Walton Centre for Neurology and Neurosurgery, Liverpool, UK; 4 Department of Neurology, Ludwig Maximilian University of Munich, Munich, Germany; 5 Department of Neurology, Liège University, Liège, Belgium; 6 Berlin NeuroImaging Center, Charité University Hospital, Berlin, Germany; 7 Clinical Affairs, electroCore Medical, Gothenburg, Sweden; 8 Scientific Medical and Clinical Affairs, electroCore LLC, Basking Ridge, USA

## Introduction

Cluster headache (CH) is a painful and debilitating disorder for which non-invasive vagus nerve stimulation (nVNS) may be a treatment option.

## Aim

Compare the efficacy of gammaCore®, a handheld nVNS device, with the standard of care (SoC) in chronic CH subjects in the randomized phase of the Prevention and Acute (PREVA) Treatment of Chronic Cluster Headache study.

## Methods

PREVA was a multicenter study comprised of 3 phases: 2-week run-in, 4-week randomized (1:1; nVNS vs SoC), and 4-week extension. Subjects randomized to nVNS delivered stimulations prophylactically twice daily (mandatory) and optionally for the rescue treatment of CH attack. The primary efficacy end point was the reduction in number of CH attacks/week during the last 2 weeks of the randomized phase versus the run-in phase. Additional end points included the proportion of subjects with >50% reduction in CH attacks/week (response rate) and rescue medication use; safety was assessed by monitoring the frequency of adverse events.

## Results

Ninety-seven subjects were randomized; data from 93 subjects (n=45 nVNS; n=48 SoC) were included in the intention-to-treat population. Number of CH attacks/week was significantly reduced in subjects treated with nVNS compared with patients treated with SoC only (-7.6 vs -2.0; P=.002). Further, significantly more nVNS- than SoC-treated subjects were considered treatment responders (34.4% vs 7.1%; P=.003). nVNS was associated with less use of rescue medications and demonstrated a favorable safety/tolerability profile.

## Conclusion

Prophylactic treatment of chronic CH with nVNS is safe and, compared with SoC, reduces frequency of CH attacks/week. Sham-controlled studies are warranted and underway to confirm these data.

Abstract submitted on behalf of the PREVA Study Investigators.

